# Deregulation of ncRNA in Neurodegenerative Disease: Focus on circRNA, lncRNA and miRNA in Amyotrophic Lateral Sclerosis

**DOI:** 10.3389/fgene.2021.784996

**Published:** 2021-12-02

**Authors:** Paola Ruffo, Claudia Strafella, Raffaella Cascella, Valerio Caputo, Francesca Luisa Conforti, Sebastiano Andò, Emiliano Giardina

**Affiliations:** ^1^ Medical Genetics Laboratory, Department of Pharmacy, Health and Nutritional Sciences, University of Calabria, Rende, Italy; ^2^ Genomic Medicine Laboratory UILDM, IRCCS Santa Lucia Foundation, Rome, Italy; ^3^ Medical Genetics Laboratory, Department of Biomedicine and Prevention, Tor Vergata University, Rome, Italy; ^4^ Centro Sanitario, University of Calabria, Arcavacata di Rende, Italy

**Keywords:** ncRNA, circRNA, lncRNA, miRNA, amyotrophic lateral sclerosis, RNA-seq, neurodegenerative disorders, NGS

## Abstract

Parallel and massive sequencing of total RNA samples derived from different samples are possible thanks to the use of NGS (Next Generation Sequencing) technologies. This allowed characterizing the transcriptomic profile of both cell and tissue populations, increasing the knowledge of the molecular pathological processes of complex diseases, such as neurodegenerative diseases (NDs). Among the NDs, Amyotrophic Lateral Sclerosis (ALS) is caused by the progressive loss of motor neurons (MNs), and, to date, the diagnosis is often made by exclusion because there is no specific symptomatologic picture. For this reason, it is important to search for biomarkers that are clinically useful for carrying out a fast and accurate diagnosis of ALS. Thanks to various studies, it has been possible to propose several molecular mechanisms associated with the disease, some of which include the action of non-coding RNA, including circRNAs, miRNAs, and lncRNAs which will be discussed in the present review. The evidence analyzed in this review highlights the importance of conducting studies to better characterize the different ncRNAs in the disease to use them as possible diagnostic, prognostic, and/or predictive biomarkers of ALS and other NDs.

## Introduction

The accessibility to plenty of data concerning transcriptional factors, non-coding RNAs (ncRNAs), RNA editing, alternative splicing represented a crucial milestone for improving the knowledge of complex disorders (Liu et al., 2017). In particular, ncRNAs include small nucleolar RNAs (snoRNAs), small interfering RNAs (siRNAs), microRNAs (miRNAs), circular RNAs (circRNAs) and long-non-coding RNAs (lncRNAs). All of them have been extensively investigated as contributing factors in various metabolic processes such as programmed cell death, development and differentiation as well as in several transcriptional process and post-transcriptional modifications (Ma et al., 2020). In general, ncRNAs have been described as significant players in biological regulatory networks that, in turn, affect different protein effectors involved in the response to specific biological stimuli or in determining the future of the cell (Wang et al., 2019). Alternative RNA splicing is a biochemical process in which introns are removed and remaining exons are bound, creating thereby different transcription isoforms of individual genes in order to increase the molecular diversity (Scotti and Swanson, 2016a; Bagyinszky et al., 2020; Elorza et al., 2021). In human cells, about 90–95% of genes are subjected to alternative splicing. Genetic mutations affecting such process can lead to the formation of abnormal transcripts or proteins with altered stability and function (Scotti and Swanson, 2016a; Elorza et al., 2021). In fact, several studies have established that mis-splicing is involved in various diseases such as cancer, muscular dystrophy, neurodegenerative diseases Ule and Blencowe, 2019; Srebrow and Kornblihtt, 2006; Scotti and Swanson, 2016b; Nik and Bowman, 2019.

NDs encompass a broad range of neurological disorders characterized by a progressive loss of neurons in specific areas of the brain or spinal cord. Although some family cases carry pathogenic mutations segregating with the disease, the etiology of NDs is often multifactorial, with the onset, progression and therapeutic response affected by a complex interaction among multiple genes, non-genetic factors and individual lifestyle (Strafella et al., 2018). GWAS studies found several SNPs correlated with the susceptibility to sporadic cases of NDs, although they are not sufficient to explain the complex phenotypes displayed by the affected individuals. In this context, the analysis of the transcriptome can add knowledge concerning the functional correlation between SNPs and clinical phenotypes (Sutherland et al., 2011). Several studies highlighted the advantages of running deep analyses of the transcriptome as a promising tool to implement the research on NDs, including the study of the role of ncRNAs and their impact on gene expression, neuronal function and viability in affected subjects. However, whether the deregulation of genes and transcriptional signatures are a cause or consequence of the onset of NDs is still a matter of debate (Costa et al., 2013).

Among NDs, Amyotrophic Lateral Sclerosis (ALS, also known as Lou Gehrig’s disease) is caused by the progressive loss of MNs, resulting in the paralysis of voluntary muscles, muscle atrophy, stiffness, fasciculation and progressive difficulty swallowing, phonation and respiratory function (Hobson et al., 2016; Harrison et al., 2018n). Sporadic ALS (sALS) is the most common form of disease, accounting for about 90% of all cases. Family ALS (fALS), instead, affects about 10% of individuals and is inherited by an autosomal dominant pattern (Chen et al., 2013). The 40–55% of familial cases are due to pathogenic mutations in disease-associated genes, among which *SOD1*, *FUS*, *TARDBP* and *C9ORF72* are the most frequently involved (Perrone Benedetta e Franc, 2020). This disease has a very rapid course and, to date, the diagnosis is often made by exclusion because there is not a specific symptomatology framework. This is the reason why, it is important to research clinically useful biomarkers addressed to make a faster and more precise diagnosis of ALS, especially in cases where there are not genetic mutations or cases of affection within the family.

It is known that metabolomics studies have allowed the identification of various metabolites related to altered pathophysiology of ALS that could represent specific biomarkers alone or in concordance, identifying a specific metabolic signature for ALS. The identification of these signatures allows the development of personalized therapy. Glutamatergic excitotoxicity, stress and the progression of energy metabolism have been discovered, thanks to differential oxidative metabolomics experiments, as key factors leading to the degeneration of MNs. Such alterations have been observed both in affected patients and in disease models strengthening their role as biomarkers (Lanznaster et al., 2018).

In addition, many recent studies have focused on the role of neurofilaments (NFs) as biomarkers in ALS. NFs are cytoskeletal proteins and their levels improve in biological fluids in proportion to the axonal degree. The work conducted by Sun et al., 2020, confirmed that neurofilament reading chain (NFL) levels are promising prognostic biomarkers for monitoring disease severity and progression of ALS. There are several research groups that have confirmed the use of NFL as specific biomarkers of the disease (Tortelli et al., 2015; Forgrave et al., 2019; Verde et al., 2019; Benatar et al., 2020).

Up to date, the molecular mechanisms of ALS are not completely understood. ALS is a complex and multifactorial disease characterized by the involvement of several pathological processes. The most characteristic pathogenic mechanisms of ALS include axonal transport dysfunctions, apoptotic mechanisms, neuroinflammation, proteins aggregation and abnormal mitochondrial function (Krokidis and Vlamos, 2018a). Over the different molecular mechanisms which have been associated with ALS in the last years, some of which include the action of non-coding RNAs, including miRNAs, lncRNAs and circRNAs (Salta and De Strooper, 2017), which will be discussed in the present review.

### The Use of RNA-SEQ Analysis for Elucidating ALS Mechanisms

The application of NGS technologies in the context of modern molecular medicine has provided many data from DNA or RNA samples, both in terms of qualitative and quantitative information, that have been essential for discovering primary and secondary molecular targets in the context of NDs. In general, the RNA-seq analysis can offer a complete and detailed analysis of the whole transcriptome and a list of Differential Expressed Genes (DEG), which are useful to understand how the different genes can be up-regulated or down-regulated in affected subjects compared to healthy controls (Costa et al., 2010). The DEG analysis can be further utilized to assess how molecular pathways are modified in pathological contexts compared to health conditions and to identify which transcriptional changes can be related to the onset and progression of specific disease conditions. Indeed, transcriptional changes have been described as a consequence of biological aging or in the etiopathogenesis of complex disorders, including NDs (Costa-Silva et al., 2017; Su et al., 2019). Today, different technologies for sequencing are available: bulk and single cells RNA-seq, Poly-A and ribo-minus RNA-seq experiments, short and long reads sequencing.

Bulk RNA-seq technologies have been extensively used to primarily study average gene expression on thousands of cells. The advent of single-cell RNA sequencing (scRNA-seq) offers unprecedented opportunities to explore gene expression profile at the single cell level leading to in-depth discoveries on the variability and dynamics of cellular expression. Currently available scRNA-seq approaches still have a major problem, as weakly expressed genes are not identified. Furthermore, since most current scRNA-seq methods primarily capture polyA + RNA, the development of protocols capable of capturing both polyA + and polyA- RNAs allows for a comprehensive investigation of coding and non-coding gene expression (Chen et al., 2019a).

Recently, RNA-seq methods, based on mRNA or ribo-minus based on NGS, are considered more accurate and comprehensive for transcriptome profiling (Wang et al., 2009). In eukaryotic cells, 80% of the total RNAs are ribosomal RNA (rRNA) while the remaining 5% are positive polyadenylate [poly (A) +] mRNA. The mRNA-seq (polyA-selected RNA-sequencing) and rmRNA-seq (ribo-minus RNA-sequencing) methods selectively remove a different set of RNA: negative poly (A) RNA and rRNA, respectively. This protocol enriches the transcripts of poly (A) + including mRNA and many non-coding RNAs and also reduces the amount of pre-mRNA. In contrast, depleted rRNA removes cytoplasmic and mitochondrial rRNA and thus includes poly (A) + mRNA, as well as non-coding RNA or protein-coding mRNA that are not polyadenylated (Chen et al., 20209). The comparison between these two RNA sequencing analyses: rmRNA-seq and mRNA-seq showed that rmRNA-seq can detect more transcripts including genes encoding proteins, ncRNA, snoRNA and snRNA, highlighting how this technology provides more in-depth data than those of mRNA-seq for the systematic profiling of transcriptomes. In particular, the rmRNA-seq method allows to obtain data on different polyA-orbimorphic transcripts such as transcripts of protein-coding genes (e.g. Histone, Heg1 and Dux), ncRNA, snoRNA, snRNA and new ncRNA. However, both technologies fail to identify a significant fraction of transcripts, considered potential NpA (non-polyA) or bimorphic transcripts, and these NpA transcripts are quite abundant in eukaryotic cells up to about 80% of the total transcribed sequences (Cui et al., 2010n).

Short-read sequencing is the method for detecting and quantifying the gene expression of the entire transcriptome. This sequencing technologies perform sequencing by synthesis (SBS) or ligation. Each strategy uses DNA polymerase or ligase enzymes for numerous strands of DNA in parallel, respectively. This method requires the identification of the newly sequenced nucleotides as they are incorporated, without interrupting the synthesis process [Genomics (26 luglio 2021), 2021]. The short-read sequencing has several advantages such as, it is cheaper and easier to implement than microarrays; generates comprehensive, high-quality data that identify quantitative expression levels well across the transcriptome; it is a robust method that exhibits high intra-platform and cross-platform correlations. However, errors may occur in the sample preparation phase and during computational analysis that negatively affects the ability to correctly identify and quantify the different expression isoforms of the gene (RNA sequencing: the teenage years, 2021). Moreover, this technology cannot sequence long stretches of DNA because the DNA strands must be fragmented and amplified before the sequencing process. A new approach is the use of long-read sequencing for obtaining the full-length sequence of the mRNA. This method allows labelling full-length cDNAs with unique molecular identifiers (UMIs), which are copied along the length of individual cDNA molecules before the preparation of a short-read RNA-seq library. Transcription isoforms can be reconstructed up to 4 kb. Comparing the two methods, long read shows much lower throughput and much higher error than short-read platforms that, in turn, show off greater fidelity given their increased use. However, long reads platoforms can capture multiple transcripts (RNA sequencing: the teenage years, 2021).

Most RNA-seq studies for ALS have been performed on mouse models or cultured cells derived from the spinal cord, brain stem and Central Nervous System (CNS) (Liu et al., 2020). ALS rodent models have been indispensable for developing hypotheses on how mutant SOD1 proteins induce MNs degeneration. In Wenting Liu et al., 2020, Single-cell RNA sequencing (scRNA-seq) has been performed on a transgenic mouse model of ALS (SOD1*G93A), in particular at level of the brain stem region. The region of the brain stem has been deliberately chosen, as it is responsible for the oral-motor functions that are strongly affected by the disease. In fact, about 25% of ALS cases is characterized by progressive bulbar paralysis and maxillary muscle strength with consequences on chewing, swallowing and loss of ability to move (Riera-Punet et al., 2018). The results of the experiments highlighted the alteration of both genes and pathways already known and related to ALS, and those specific to the anatomical area of interest, such as the transport of toxins in the ependymal cells of the brainstem and the response to organophosphate in Schwann cells. Subsequently, the differentially expressed genes of the animal model were compared with the human GWAS highlighting an overlap of the two and, therefore, emphasizing that the discoveries made on murine models may be relevant to human disease (Liu et al., 2020). In another study, the identification of transcriptional changes and a high number of DEG confirmed the involvement of different types of glial cells in ALS. Animal models carrying *SOD1* mutation allowed identifying the involvement of the oligodendrocytes in the pathology, showing altered neurogenesis and nuclear envelope formation (Kang et al., 2013). At level of microglia, the immune pathway resulted to be altered, supporting the thesis that the progression of the disease is driven by changes affecting the immune-inflammatory response. Astrocytes and Schwann cells showed a significant number of DEGs and relevant transcriptomic alterations. Altogether, these evidences allow understanding that different types of cell play a specific role in the pathology that are worth to be clarified in order to devise targeted treatments. In ependymal cells, the presence of the genetic variant in *SOD1* is directly correlated in toxin transport and cell differentiation (Liu et al., 2020).

Other studies have been conducted on SALS post-mortem cortex samples and allowing distinctions into molecular subtypes characterized by different combinations of genes and pathways deregulated (La Cognata et al., 2021). The existence of distinct molecular subtypes of ALS have also been highlighted in other works (Aronica et al., 2015). Thanks to these discoveries, it is possible to think of designing targeted and effective therapies specific to each patient. The study and evaluation of post-mortem brain tissue RNA samples reveal pathogenetic mechanisms at the end-stage of the disease and do not clarify whether the transcriptional differences are a cause or a consequence of the disease process. In this context, the use of iPSC (induced pluripotent stem cells) derived from ALS patients has provided important insights into the pathophysiology of the disease, encouraging researchers to consult the molecular heterogeneity of ALS and follow the course of degeneration.

The study conducted by Kiskinis et al., 2014 involved a combined approach of stem cell reprogramming and differentiation with genome engineering and RNA-seq to identify transcriptional and functional changes induced by the *SOD1*-A4V mutation in human MNs. The study demonstrates that the *SOD1*-A4V missense mutation causes a proapoptotic phenotype in cultured human NMs, limiting their long-term survival. Thanks to the use of RNA-seq technology, the transcriptional differences between human *SOD1* +/A4V and control MNs have been defined. The results show that MNs derived from patient-specific iPSC displayed disease hallmarks, such as defects in mitochondrial morphology and transport, oxidative and ER-related stress, and an activated UPR (unfolded protein response). Subsequent functional studies demonstrated that these perturbed pathways are consequences dependent on the presence of the *SOD1* A4V mutation (Kiskinis et al., 2014). Strongly altered signalling pathways in iPSC with *SOD1* mutation, were also found by Bhinge et al., 2017. In this study, iPSC lines were generated by correcting the point mutation in *SOD1* using CRISPR-Cas9 genome editing technology, in order to exclude the possibility of observing phenotypic differences due to genetic variation in iPSC lines. By comparing the observed phenotypes with those arising from post mortem tissues of patients with the disease or in rodent models, it was confirmed that the *in vitro* model reflects specific aspects of the disease. The analysis conducted with RNA-seq identified several pathways commonly dysregulated in ALS-affected MNs, such as the activation of cell cycle genes and p53 in *SOD1*-mutant MNs. Furthermore, pharmacological inhibition of the upregulated pathways, it was possible assisting to the activation of the AP1 pathway, through MAPK signalling, with consequent neurodegeneration of MNs. Further studies are needed to elucidate the mechanisms of neurodegeneration and also to provide phenotypic screens for searching new molecular targets (Bhinge et al., 2017).

Another RNA-seq analysis was conducted on peripheral blood mononuclear cells (PBMC) from sporadic and mutated patients with ALS (mutations in *FUS*, *TARDBP*, *SOD1* and *VCP* genes) and healthy controls allowing the characterization and comparison of the entire transcriptome of the PBMC content, both in terms of coding and non-coding RNAs. The aim of this work was the creation of a dataset for RNA profiling in ALS using a tissue that is easy to collect, manage and store (Zucca et al., 2019).

Spatial transcriptomics (ST) generates quantitative transcriptome RNA sequencing data through polyadenylated RNA capture on spatially bar-coded DNA capture probe arrays. In the study conducted by Miniati et al., 2019, ST is applied to spatially profile gene expression in lumbar spinal cord tissue sections from *SOD1*-G93A (ALS) and *SOD1*-WT (control) mice at presymptomatic time points, onset, symptomatic and end-stage. In addition, this technique has been used to profile gene expression in tissue sections of the accumulated spinal cord and post mortem cervical from sporadic lumbar or bulbar onset ALS patients. From the results obtained, it has been possible to distinguish the differences between the populations of microglia and astrocytes during the onset of the disease and the gene expression changes of the different transcriptional pathways. Furthermore, thanks to the procedure implemented, it has been possible to draw deductions from mouse models and then testing them in clinical samples (Spatiotemporal dynamics, 2021).

### RNA Molecules as Biomarkers in ALS

A hallmark of NDs is protein aggregation and alterations in RNA metabolism (Kinoshita et al., 2021). The alterations affect all levels of gene regulation, from RNA synthesis to degradation, and have been associated with specific alterations in RNA-binding proteins (RBPs) and non-coding RNAs. These ncRNAs are stable constructs in body fluids where their presence and potential could serve as likely non-invasive biomarkers of NDs, including ALS (Competing EndogenousA, 2021). Among non-coding RNAs, the following subsections of the review will discuss the studies concerning the role of miRNAs, lncRNAs and circRNAs in ALS physiopathology and their potential application as disease biomarkers.

### miRNAs

**FIGURE 1:**
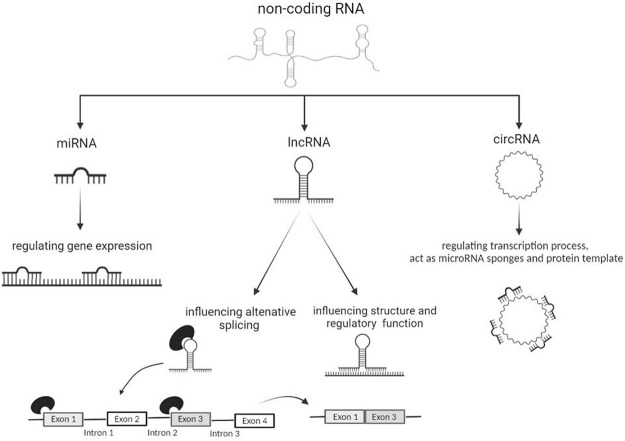
The actions of miRNAs, lncRNAs and circRNAs on different molecular processes.

MicroRNAs (miRNAs) are small oligonucleotide sequences (about 19–22 base pairs) of single-stranded non-coding RNA, which play a crucial function by regulating gene expression at post-transcriptional level (Figure 1). The action of miRNAs has been suggested as a mechanism to regulate neuroinflammation in different NDs including ALS (Benigni et al., 2016; Bai et al., 2017a; Wang et al., 2020; Strafella et al., 2021a; Strafella et al., 2021b).

Several studies highlighted how altered biogenesis and expression of miRNAs can be responsible of the degeneration of spinal motor neurons, both in fALS and sALS (Olejniczak et al., 2018; MicroAs in amyotrophic, 2021; Vaz et al., 20217; Krokidis and Vlamos, 2018b). In addition, miRNAs have been found to exert a function in neuronal inflammation in ALS (Emde et al., 2015). Numerous works showed that miRNAs in affected subjects can cross the blood-brain barrier to reach the bloodstream and, thereby, could be utilized as biomarkers of disease (Saucier et al., 2019; Liu et al., 2021). Highly deregulated miRNAs have been associated with different degrees of disease progression. miR-151a-5p, miR-199a-5p and miR-423-3p were seen to be down-regulated in affected subjects, whereas miR-338-3p, miR-206 and miR-133a appeared up-regulated. Moreover, up-regulation of miR-199a-5p, miR-206, miR-133a correlated with a better prognosis and a slower course of disease (Dobrowolny et al., 2021). miR-338-3p is involved in ALS pathogenesis, not only in tissues directly related to the disease but also in the peripheral tissues such as blood, helping to evaluate the potential of such miRNAs as a novel class of genetic blood marker for sALS (De Felice et al., 2014). miR-199a-5p has been involved in the initial phase of ALS and has been found significantly down-regulated in the last phase of disease. In contrast, miR-206 has been supposed to mitigate the progression of the disease by enhancing the regeneration of the joints at neuromuscular level (Dobrowolny et al., 2021). miR-335-5p was found to be downregulated in ALS patient serum, increasing oxidative stress, inhibiting caspase 3/7 apoptotic pathway, and deregulating neuronal degeneration (De Luna et al., 2020).

Recent studies have highlighted the presence of connections between miRNAs and RBPs, such as TDP-43 and FUS, with essential regulatory complexes such as Drosha in the nucleus and Dicer in the cytoplasm. Drosha complexes with DGCR8 have also been associated with TDP-43, suggesting a more complex dynamics than miRNAs and protein-related pathologies, especially in MNs. FUS gene localizes together with TDP-43 in the Drosha nuclear complex and the direct binding of FUS to the nascent pri-miRNAs allows to recruit Drosha to transcriptionally active sites for further processing of the pri-miRNAs themselves. Furthermore, FUS has been shown to promote gene silencing through direct binding to certain miRNA and mRNA targets. Mutations in this gene also impair the function of the AGO2 protein in the miRNA-induced silencing complex (miRISC) (Pham et al., 2020).

In subjects affected by fALS and sALS carrying *SOD1* mutation, the up-regulation of miR-129-5p revealed a direct action against the *ELAVL4* gene transcript (*Elav-Like RNA-Binding Protein 4*, 1p34, OMIM #168360). It is interesting to note that this transcript encodes HuD protein, which binds RNA and is mainly expressed at the neuronal level, where it is involved in many molecular processes, such as the control of neuronal life by promoting the translation of the mRNAs involved in axonal and neuronal formation. Experimental studies have shown how HuD is down-regulated in *SOD1* mutated samples; miR-129-5p acts positively against HuD by increasing its expression and preventing this protein from being degraded (Loffreda et al., 2020). Furthermore, *ELAVL4* have also been found deregulated in *in vitro* models carrying mutations in *FUS* gene downstream miR-375 deregulation (De Santis et al., 2017; Comparative interactomic, 2021; MutantS andL4 (H, 2021; Dell'Orco et al., 2021).

### lncRNAs

Long non-coding RNAs (lncRNAs) consist of more than 200 nucleotides and are involved in the regulation of several biological functions (Ma et al., 2020). lncRNAs are characterized by a tissue-specific expression and play a modulatory role in the CNS by influencing epigenetic processes, post-translational and transcriptional regulation, alternative splicing and cell cycle (Yuan et al., 2020) (Figure 1). These constructs are well known to be associated with the pathogenesis of many NDs and a more detailed understanding of them could identify them as specific biomarkers of disease (Bhattacharyya et al., 2021).

RNA-seq analyses of sALS and fALS patients revealed the presence of lncRNAs differentially expressed both in blood mononuclear cells and in the spinal cord (Gagliardi et al., 2018). Several lncRNAs have been found both in MNs and in peripheral blood such as *NEAT1* (*Nuclear Paraspeckle Assembly Transcript 1*, 11q13.1, OMIM #612769), *MALAT1* (*Metastasis-Associated Lung Adenocarcinoma Transcript 1,* 11q13.1, OMIM # 607924) and *MEG3* (*Maternally Expressed Gene 3*, 14q32.2, OMIM # 605636). The RBPs associated with ALS have been shown to interact with NEAT1 and regulate its expression. Several studies have shown how NEAT1 binds FUS and TDP-43 by increasing the frequency by which paraspeckles (i.e.subnuclear bodies located in the interchromatin space of cells) are formed at the nuclear level and in MNs (Vangoor et al., 2021). Paraspeckles play a fundamental role in the control of gene expression thanks to the nuclear retention of modified RNA. Through this mechanism, paraspeckles can control gene expression during many cellular processes including differentiation, viral infection and stress responses (Fox and Lamond, 2010), as well as exert anti-apoptotic activity. An increase in the early stages of ALS could be related to an increase in the survival of MNs. Many studies have shown that RBP mutations influence the formation of paraspeckles leading to a more aggressive phenotype. Indeed, mutations in *FUS* result in lower paraspeckle production and dysregulation of *NEAT1* transcription (Vangoor et al., 2021) (Gagliardi et al., 2018). A study by Suzuki et al., 2019 correlated *NEAT1* expression to defects in the genesis of paraspeckle that cause neurodegeneration and neuronal death in ALS (Suzuki et al., 2019). Many disease-related genes can give rise to sense or anti-sense RNA that are then translated into proteins but play the role of lncRNAs. The anti-sense transcripts perform several functions such as the activation of RNA interference caused by the formation of double-stranded RNA created by the union of single complementary strands; transcriptional interference caused by the displacement of transcription factors in the promoter region; epigenetic regulation mediated by the recruitment of chromatin remodelling factors. Based on these different roles, the use of sensory oligonucleotides as a targeted therapy could be hypothesized. The *ATXN2* gene (*Ataxin 2*, 12q.24.1, OMIM #601517) has been associated with ALS because of its interaction with FUS and TDP-43, by which it has been supposed to contribute to the disease pathogenesis. Mutations in *ATXN2* can cause the production of antisense transcripts that are present in the tissues of ALS patients. *MALAT1* (OMIM * 607924) has shown a high affinity for TDP-43, whose binding result in a subsequent increased expression, whereas *MEG3* (OMIM * 605636) has been found down-regulated and displayed a lower binding of TDP-43. The different interactions of *FUS* and TDP-43 with different lncRNAs could be associated with degeneration of MNs in ALS, with a mislocalization of the genes themselves and impact on the distribution of MNs (Vangoor et al., 2021).

In Y. Yu, et al., 2021, six differentially-regulated lncRNAs emerged in peripheral leukocytes between sALS patient and control. Of them, lnc-ABCA12-3: 1, lnc-DYRK2-7: 1 and lnc-POTEM-4: 7 have been proposed as sALS markers. In particular, Lnc-DYRK2-7: 1 and lnc-POTEM-4: 7 have been specifically down-regulated in affected subjects compared to healthy controls (Yu et al., 2021).

### circRNAs

RNA sequencing technology is currently the only method capable of providing a complete landscape of circRNAs throughout the body and in specific tissue areas (Philips et al., 2020). In particular, ribo-minus RNA-seq, has allowed identifying new change in circRNAs expression and also investigate the roles of these circRNAs in the condition of interest (Cooper et al., 2018). circRNAs are very stable regulating molecules within the cell as they are resistant to the action of exonucleases (Chen, 2016; Xie et al., 2017). circRNAs can act as transcriptional regulators, miRNA sponges and protein template, although emerging evidence described them as protein decoys, scaffolds and recruiters (Zhou et al., 2020) (Figure 1). circRNAs are actively involved in the formation of muscle tissues (Legnini et al., 2017); synaptic formation and activity (Chen et al., 2019b); control of neuronal gene expression; neuronal differentiation and development of the CNS. circRNAs are ubiquitously present in many cell types, although they are particularly enriched at neuronal level (Zhou et al., 2020; Legnini et al., 2017; Chen et al., 2019b; D’Ambra et al., 2019).

Concerning circRNAs and ALS, a recent paper (Dolinar’s article et al., 2019) presented the first differential expression analysis of circRNAs in patients with ALS diagnosis (Dolinar et al., 2019). The analysis was performed on leukocytes, considering that blood is an easily accessible biological source and therefore it is more suitable for diagnostic use.

The experiment results indicate hsa_circ_0063411, hsa_circ_0023919, hsa_circ_0088036 as potential blood biomarkers for ALS. Specifically, hsa_circ_0023919 is located within *PICALM* gene (*Phosphatidylinositol-binding Clathrin Assembly Protein*, 11q14.2, OMIM #603025) and presents two link sites for hsa-miR-9, which appeared up-regulated in patients with sALS (Vrabec et al., 2018). However, further studies are necessary to test the potential association between hsa_circ_0023919, hsa-miR-9 and the disease (Dolinar et al., 2019). Concerning hsa_circ_0063411, more in-depth and targeted studies are needed to evaluate the possible relationship with hsa-miR-647, given the presence of a link site for this miR on the circRNAs. There is no biological evidence regarding this circRNAs but its ligand, hsa-miR-647, has been found down-regulated in ALS patients (Bai et al., 2017b). The hsa_circ_0088036 is found within *SUSD1* gene (*Sushi domain containing 1*, 9q31.3, OMIM #607723), which has been associated with ALS (Dolinar et al., 2019). Levels of such circRNAs have been found up-regulated in ALS patients as well as in patients suffering from Rheumatoid Arthritis, for which hsa_circ_0088036 is already considered a biomarker (Group Therapy for Schizop, 2020d). Since these two disorders share some common mechanisms, it would be interesting to further investigate the role of hsa_circ_0088036 in ALS as well. In the study conducted by Dolinar et al., 2019, hsa_circ_0088036 and hsa_circ_0023919 were negatively associated with the age of onset of disease, whereas hsa_circ_0063411 was negatively associated with the duration of the disease and survival (Dolinar et al., 2019). This work has laid the foundations for considering circRNAs as diagnostic biomarkers, although further studies are needed to clarify their association with the pathology.

A direct role in regulating circRNAs production has been shown by *FUS* gene. In particular, the RNA-binding protein FUS has been identified as a novel regulator of circRNAs production and also a key player in controlling the expression of these transcripts in mouse MNs. In these *in vitro*-derived MNs there is a high number of circRNAs and a specific subclass is influenced by the levels of *FUS*, which can enhance or repress the back-slicing reaction. The analysis of the subcellular localization identified nuclear circRNAs species entirely derived from exonic sequences. The fact that *FUS* is involved in circRNAs biogenesis is important not only to elucidate its role in this process, but also to link the function of circRNAs to neurodegenerative processes (Errichelli et al., 2017).

## Conclusion and Future Prospective

**FIGURE 2:**
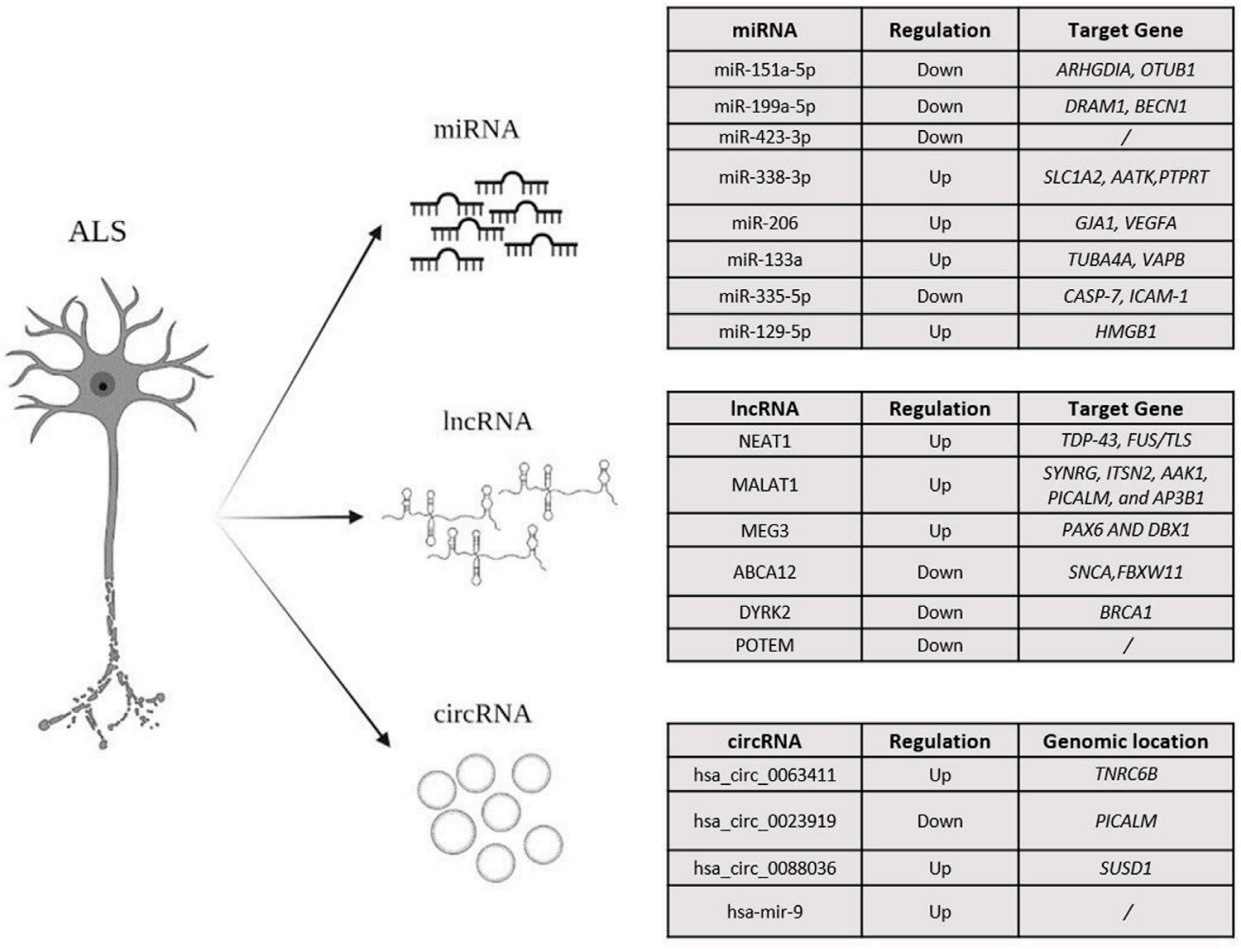
An overview of the miRNA, lncRNA and circRNAs, mainly investigated in ALS.

The availability of NGS technologies allowed the massive and parallel sequencing of total RNA samples derived from a range of specimens. This enabled the characterization of the transcriptomic profile of several cell populations and tissues, providing a more detailed and accurate overview of complex traits and phenotypes. Several studies and experiments showed that the alteration of RNA metabolism, function, structure and localization of both coding and non-coding RNAs are involved in the onset and progression of ALS (Zucca et al., 2019). The use of RNA-seq analysis highlighted the existence of a wide class of ncRNAs (Figure 2), whose investigation is providing more and more insightful clues for improving the knowledge of disease and addressing the forthcoming research efforts towards the development of more effective clinical treatments and diagnostic protocols.

In this context, it is important to note that recent studies showed that lncRNA and mRNA can compete for binding to miRNAs and this interaction can play a role in different diseases, including ALS (Liu et al., 2021). The potential cross-talk between miRNA-lncRNA-mRNA can be investigated by means of competitive endogenous RNA (ceRNA) network analysis. This model allows assessing how the different ncRNAs can interact together and affect the molecular pathogenesis of disease (Sardina et al., 2017). On this subject, Liu et al. (2021) created a ceRNA network with the aim of making a molecular characterization of the ALS development and providing promising targets for clinical treatment. The study made it possible to understand that the regulation of MALAT1 plays an important role in the development of the disease. Furthermore, the *SYNRG*, *ITSN2*, *AAK1*, *PICALM* and *AP3B1* genes associated with ALS and regulated by MALAT1, have been found to play an important role in the pathogenesis of ALS. In particular, the discovery of the association between *AAK1* gene and ALS is of considerable interest, although further research is necessary to understand the correlation of this gene and the other ones with the disease (Liu et al., 2021).

In conclusion, the above-discussed recent findings emphasized that the future studies should be tailored to better characterize the pathophysiological role of the different ncRNAs and ceRNA networks in the disease, aiming to employ them as possible prognostic, predictive and/or diagnostic biomarkers for ALS and other NDs.
